# Sequential bacterial sampling of the midline incision in horses undergoing exploratory laparotomy

**DOI:** 10.1111/evj.12958

**Published:** 2018-05-17

**Authors:** C. M. Isgren, S. E. Salem, N. B. Townsend, D. Timofte, T. W. Maddox, D. C. Archer

**Affiliations:** ^1^ Department of Epidemiology and Population Health Institute of Infection and Global Health, University of Liverpool Leahurst UK; ^2^ Department of Equine Clinical Science Institute of Veterinary Science, University of Liverpool Leahurst UK; ^3^ Department of Surgery Faculty of Veterinary Medicine Zagazig University Zakazik Egypt; ^4^ Three Counties Equine Hospital Stratford Bridge Ripple Tewkesbury UK; ^5^ Institute of Veterinary Science University of Liverpool Leahurst UK; ^6^ Institute of Infection and Global Health, University of Liverpool Leahurst UK; ^7^ Department of Musculoskeletal Biology Institute of Ageing and Chronic Disease, University of Liverpool Leahurst UK

**Keywords:** horse, colic, surgical site infection, bacterial culture, drug‐resistant, antimicrobial

## Abstract

**Background:**

There is limited information about bacterial isolates that are present on the equine midline incision during and following exploratory laparotomy.

**Objectives:**

To investigate the bacterial species cultured from the ventral midline pre‐, intra‐ and post‐ laparotomy, whether particular bacterial isolates are associated with the development of surgical site infections (SSIs) and to report the antimicrobial resistance phenotypes of these isolates.

**Study design:**

Prospective cohort study.

**Methods:**

The ventral midline of 31 horses undergoing exploratory laparotomy was sampled for bacterial culture at set time‐points pre, intra and post‐operatively. Inclusion criteria were that horses must have undergone exploratory laparotomy within 90 min of the initial colic examination upon hospital admission and must not have been placed in a stable prior to surgery. SSI was defined as any purulent or serous discharge from the laparotomy incision of >24 h duration.

**Results:**

Seven horses (22.6%) developed a SSI. None of the variables tested were associated with the altered risk of SSI. The prevalence of a positive bacterial culture from the incision increased progressively over time and a variety of bacteria were isolated. A positive intra‐operative culture was not a predictor of SSI; and when a SSI did occur, it was due to a different bacterial isolate. MRSA and ESBL‐producers were identified in the post‐operative period in one and four different horses respectively, but none of these developed a SSI.

**Main limitations:**

Sampling was limited to hospitalisation and no culture results were available for horses developing SSI following hospital discharge**.**

**Conclusions:**

A variety of bacterial species may be isolated from equine laparotomy incisions peri‐operatively without development of SSI. SSI does not appear to be solely related to bacterial contamination of the incision peri‐operatively and other mechanisms such as bacteraemia merit further investigation.

## Introduction

Surgical site infection (SSI) of the abdominal incision following exploratory laparotomy occurs in 10–37% of horses 1 and is a cause of significant post‐operative morbidity. Incisional hernia formation is 4–9 times more likely to develop in horses with SSI, which may necessitate further surgery with associated risks to the horse and costs to the owner 2, 3. Bacterial culture and susceptibility testing results from SSIs often reveal multiple bacterial isolates, many of which are resistant to common antimicrobials 4. The development of SSI is a multifactorial process and is likely to be influenced by the bacterial load of the site and local factors, as well as host defences 5, 6. A number of patient and procedure‐related factors have been found to influence the likelihood of development of incisional SSIs following abdominal surgery in horses 4. Variation between studies is likely due to inconsistencies in definitions of SSI used and the duration of follow‐up 7.

Post‐operative protection of the incision, including the use of a sewn on stent bandage 8 or an abdominal bandage 9 has been reported to reduce the rate of SSI. As these interventions physically separate the incision from the surrounding environment, it is plausible that they reduce SSI by minimising contamination by environmental bacteria during anaesthetic recovery and the early post‐operative period. Studies investigating the source and timing of contamination of surgical incisions in humans and horses have reported variable results. A correlation behind high bacterial load of the incision on the second day following surgery and subsequent development of SSI was reported in a study of human lower limb vascular surgery 10. In horses, a prospective pilot study, reporting on sequential midline culture following exploratory laparotomy similarly demonstrated that SSI was associated with significant bacterial growth on the midline incision after anaesthetic recovery and at 24 h post‐operatively 11. In the latter study, no further sampling was performed beyond 24 h post‐operatively and SSI was defined as presence of any incisional drainage, without obtaining bacterial culture of the draining material. Two separate studies have found intraoperative culture results to be poor predictors of SSI 12, 13.

No published study has reported sequential cultures of the abdominal incision pre‐, intra‐, and post‐laparotomy. The aims of the current study were to determine the bacterial species cultured from the ventral midline incision peri‐operatively, to investigate whether particular bacterial isolates were associated with development of SSI and to report the antimicrobial resistance phenotypes of the bacteria cultured. Our hypotheses were that horses with a positive culture either intra‐operatively or immediately following recovery from anaesthesia would be more likely to develop SSI than those with a negative culture, and that the bacterial species isolated from horses that developed an SSI would be the same as those cultured previously from the incision in the same horse.

## Materials and methods

### Study design and case selection

**Figure 1:**
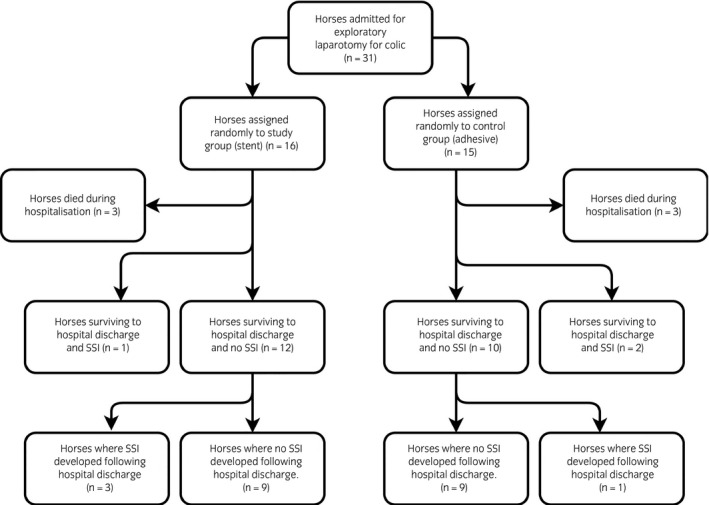
Flow chart to show recruitment of horses into, and their progression through the study.

Horses that underwent exploratory laparotomy for treatment of colic at The University of Liverpool, Equine Hospital over a 12‐month period (August 2014–July 2015) were recruited. Horses were selected by convenience sampling, and included those undergoing surgery when the first author was available to implement a rigid standardised sampling protocol. Horses were only included in the study if they underwent exploratory laparotomy within 90 min of initial examination performed at hospital admission and must not have been stabled prior to surgery (to avoid potential contamination of the ventral midline with resident hospital bacterial populations prior to sampling). Follow‐up beyond hospital discharge was performed via a telephone questionnaire by the first author (Supplementary Item 1) with the owner at least 3 months after the horse was discharged. A flow chart to show recruitment of horses into, and their progression through the study is shown in Fig 1. Horses that had undergone exploratory laparotomy in the previous 12 months were excluded, as these horses may be at increased risk of developing incisional complications 2. An adhesive drape^a^ designed for laparotomy was used in all horses to minimise contamination of the incision during surgery. The midline incision site was swabbed at the following sites and time points: 1) immediately following clipping but prior to aseptic preparation of the ventral midline (4% chlorhexidine gluconate scrub for at least 10 min followed by 2% chlorhexidine gluconate and 70% isopropyl alcohol mixture solution), 2) following aseptic preparation but immediately before the first incision was made, 3) following closure of linea alba but before lavage of the incision, using sterile polyionic fluids, 4) following closure of the skin, 5) immediately following recovery from general anaesthesia after removal of the protective dressing, and 6) every 48 h during hospitalisation when the abdominal dressing was changed. The incision was not cleaned at dressing changes unless it was exposed or if there was discharge present. If there was discharge present, the incision was cleaned in a sterile fashion with 1% hydrogen peroxide solution and if incision was exposed it was cleaned with 0.05% chlorhexidine gluconate 14. A charcoal medium transport swab^b^ was used to sample the surgical site in a sterile fashion prior to cleaning of the incision. Bacterial culture consisted of direct plating onto 5% sheep blood agar and plates were then incubated aerobically and anaerobically for 2–7 days at 37.0°C. Quantification of bacterial growth was performed by assessing the amount of bacterial growth obtained within the four quadrants of the culture plates. Results were reported as pure growth (heavy, moderate or light) or as mixed growth with the isolated organisms enumerated according to the weight of growth (from heavy to light). Microorganisms isolated from positive cultures were identified using API kits^c^ and GNID and GPID Sensititre Identification plates^d^. Susceptibility testing was determined using standard disc diffusion technique on Mueller–Hinton agar according to the Clinical and Laboratory Standards Institute (CLSI) 15 guidelines. Antimicrobials included ampicillin (10 μg), ceftiofur (30 μg), trimethoprim/sulfamethoxazole (25 μg), enrofloxacin (5 μg), erythromycin (30 μg), gentamicin (10 μg), oxacillin (1 μg), metronidazole (5 μg), neomycin (10 μg), oxytetracycline (30 μg), penicillin G (1.5 units) and streptomycin (25 μg) (all media and antimicrobial discs were from Oxoid)^e^. Interpretation of susceptibility results was performed according to CLSI 15 and CLSI for animals 16 for enrofloxacin and ceftiofur.

This study was part of a larger prospective randomised controlled trial investigating the use of a stent to protect the incision during recovery and all horses were randomly assigned to one of two groups. In the first group (Group A), an adhesive dressing^f^ followed by an adhesive impervious sheet^f^ were applied at the end of surgery. Once in the recovery room, an abdominal wrap^g^ was placed. In the second group (Group B), protection of the incision was identical to the first group but an equine stent bandage^a^ was sutured in place instead of the adhesive dressing. Digital images of the incision following recovery were used to verify if the incision had been fully protected, partially protected or fully exposed during anaesthetic recovery. Post Anaesthetic Recovery Quality Scores (1–5) were obtained for each horse 17. Following anaesthetic recovery, all abdominal protective layers were removed and a standard abdominal bandage was placed in a sterile fashion. This consisted of a sterile absorptive contact layer^f^ placed on the incision followed by application of an elastic cohesive dressing^h^ with an elastic adhesive dressing^h^ securing the cranial edge of the bandage to prevent slipping. The abdominal bandages were changed every 48 h until discharge from the hospital when it was either removed completely or a new bandage was placed which the owner was instructed to remove 48 h later. Digital images of the incision and the dressing were obtained at subsequent abdominal dressing changes (q. 48 h). The images were recorded and were independently assessed by three blinded observers (two interns and one resident) at the end of the study. SSI was defined as any purulent or serous discharge from the laparotomy incision of >24 h duration.

Standard post‐operative medical therapy consisted of flunixin meglumine administration for at least 48 h (1.1 mg/kg i.v. q.12 h). All horses received antimicrobials immediately prior to induction of anaesthesia, either procaine penicillin (12 mg/kg i.m. q.12 h) alone or in combination with gentamicin (6.6 mg/kg i.v. q.24 h) for 3–5 days based on clinician preference and procedure performed. Horses considered to be at high risk of developing postoperative ileus (POI) were placed on a lidocaine infusion (1.3 mg/kg bolus given over 15 min followed by 0.05 mg/kg/min continuous rate infusion [CRI]) 18 and polymixin B (5000 iu/kg i.v. q. 12 h) was administered to some horses that were considered likely to develop endotoxaemia/systemic inflammatory response syndrome (SIRS) post‐operatively based on clinician preference. In horses that developed a SSI, the abdominal bandage was changed daily and the incision was cleaned with 1% hydrogen peroxide at each bandage change 19.

### Data analysis

Summary statistics were generated for all the variables recorded and data were examined for missing or outlying data points. Only culture results obtained in the first six sampling occasions were considered in the statistical analysis due to concerns that post‐operative recolonisation by normal skin flora would influence the analyses. A Fisher's exact test was used to explore the association between categorical variables including positive culture at time point 1–6 (yes/no), degree of growth (none, light, moderate, heavy), growth of drug resistant (DR, defined as resistance to at least one class of antimicrobials) or multidrug resistant (MDR, defined as resistance to at least one agent in three or more antimicrobial classes) isolates 20, sex, use of a stent, performing an enterotomy, if the incision remained protected during recovery, duration and type of systemic antimicrobials and SSI. The outcome of interest was SSI (dichotomous variable). A Fisher's exact test was also used to test associations between performing an enterotomy or anastomosis and a positive intraoperative culture; recovery score and a positive culture following recovery; recovery score and likelihood of the incision remaining protected at the end of anaesthetic recovery. The outcome of interest for these analyses were positive intraoperative culture, positive culture following recovery or the incision remaining protected at the end of recovery, respectively. A Wilcoxon rank sum test was used to explore the association between continuous variables (age, weight, heart rate, packed cell volume, and total plasma protein, blood lactate on arrival) and SSI. All analyses were performed using R software version 3.3.0 21 with R Base package. A P value of ≤0.05 was regarded as significant.

## Results

**Figure 2:**
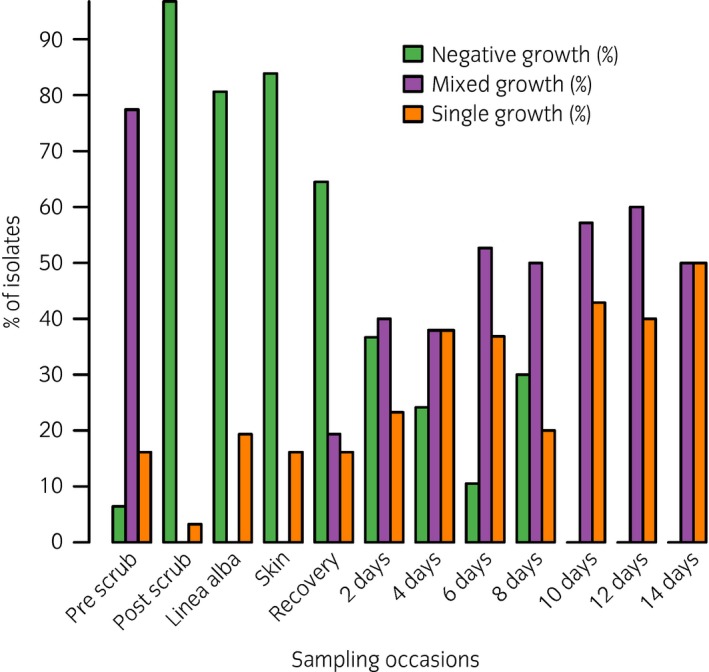
Proportion of horses with a negative, single or mixed growth culture at different time points from prior to aseptic preparation of midline until discharge from hospital or death. Mixed growth were those where more than one type of bacterial isolate were identified.

A total of 257 samples were obtained from 31 horses that fulfilled the inclusion criteria. The median number of sampling time points was 8 (interquartile range 7–9). Procaine penicillin alone was administered in eight horses (25.8%) whilst penicillin and gentamicin were administered in 23 horses (74.2%). Following clipping of the hair coat and prior to aseptic preparation of the ventral midline, a positive bacterial culture was obtained in 29 horses (93.5%). The most commonly isolated bacterial species at this time point were *Bacillus* spp. (22.9%) (16/70 isolates) followed by *Staphylococcus* spp. (14.3%) (10/70 isolates) and *Enterococcus* spp. (10%) (7/70 isolates). The proportion of horses with a negative, single or mixed growth culture at each time point is shown in Fig 2 and the summary of bacteriological results for the cohort is shown in Table 1. Following aseptic preparation of the surgical site a positive culture (*Enterococcus* spp.) was obtained from one horse (3.2%) while no bacteria were cultured from the remaining horses.

**Table 1:** A summary of bacteriological examination results of collected samples (257 samples)

Sample time point	No. of horses sampled	No. (%) of positive culture	No. (%) of mixed growth	No. (%) of single growth	Most common bacterial species isolated	No. of DR/MDR isolates
1	31	29 (93.5)	24 (82.8)	5 (17.2)	*Acinetobacter, Bacillus, Staphylococcus*	3
2	31	1 (3.2)	0 (0)	1 (100)	*Enterococcus*	0
3	31	6 (19.4)	0 (0)	6 (%)	*E. coli, Staphylococcus, Enterococcus*	1
4	31	5 (16.1)	0 (0)	5 (%)	*Staphylococcus, E. coli, Bacillus*	1
5	31	11 (35.5)	6 (54.5)	5 (45.5)	*Acinetobacter, Staphylococcus*	6
6	30	19 (63.3)	12 (63.2)	7 (36.8)	*Staphylococcus, E. coli, Streptococcus*	26
7	29	22 (75.9)	11 (50.0)	11 (50.0)	*Staphylococcus, Acinetobacter*	31
8	19	17 (89.5)	10 (58.8)	7 (41.2)	*Staphylococcus, Enterococcus*	26
9	10	7 (70.0)	5 (71.4)	2 (28.6)	*Staphylococcus, Enterococcus, E. coli*	9
10	7	7 (100)	4 (57.1)	3 (42.9)	*Staphylococcus, Enterococcus*	8
11	5	5 (100)	3 (60.5)	2 (40.0)	*Staphylococcus, E. coli*	9
12	2	2 (100)	1 (50.0)	1 (50.0)	*Staphylococcus, E. coli*	4

Sampling time point: 1) immediately following clipping but prior to aseptic preparation of the ventral midline; 2) following aseptic preparation but immediately before first incision was made; 3) following closure of linea alba but before lavage of the incision, using sterile polyionic fluids; 4) following closure of the skin; 5) immediately following recovery from general anaesthesia after removal of the protective dressing; and 6)–12) every 48 h during hospitalisation when the abdominal dressing was changed. DR, drug resistance (resistance to ≥1 class of antimicrobials); MDR, multi‐drug resistance (resistance to ≥3 classes of antimicrobials).

**Figure 3:**
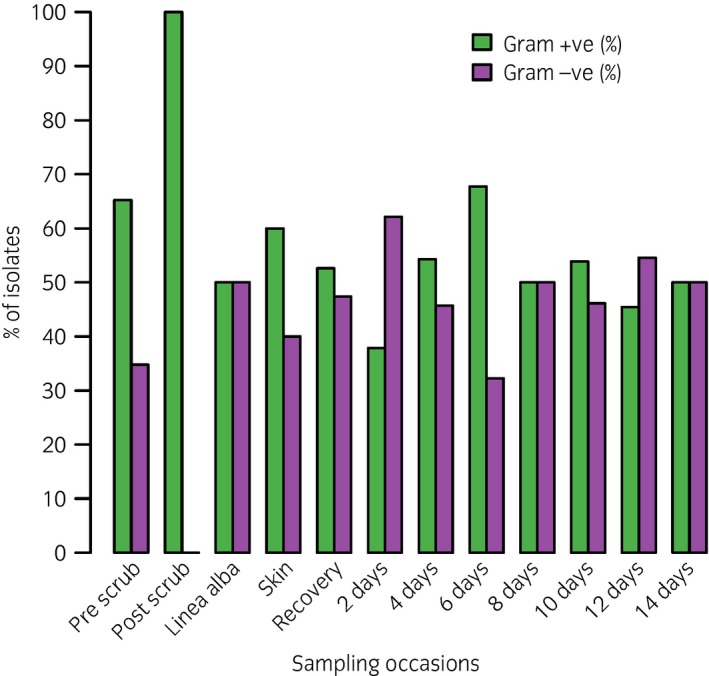
Proportion of Gram‐positive (G+ve) and Gram‐negative (G−ve) isolates at different time points from prior to aseptic preparation of midline until discharge from hospital or death.

An enterotomy or anastomosis was performed in 22 horses (70%) and no bacteria were cultured from the linea alba or skin immediately following incisional closure in 16 of these horses (73%). The positive cultures obtained in the remaining six of these horses (27%) consisted of *Enterococcus* sp. (n = 1), *Bacillus* sp. (n = 1), *Coryneform* sp. (n = 1), *Mannheimia* sp. (n = 1), *Escherichia coli* (n = 1) and *Empedobacter brevis* (n = 1). In the nine horses where no enterotomy or anastomosis was performed, no bacteria were cultured from the linea alba or skin immediately following closure in six horses (67%). The positive culture obtained in the remaining three horses (33%) consisted of *Staphylococcus* spp. (n = 2) and a Gram‐negative cocci (n = 1). The proportion of Gram‐positive and Gram‐negative isolates at each time point is shown in Fig 3. There was no association between performing an enterotomy (P = 1) or anastomosis (P = 0.7) and a positive intraoperative culture.

A positive culture following linea alba closure was found in six horses (19.3%) and there was an equal distribution of Gram‐positive (*Enterococcus* sp., *Staphylococcus* sp. *and Corynebacterium* sp.) and Gram‐negative (*E. coli*,* Acinetobacter* sp. and *Mannheimia* sp.) bacteria. A positive culture was obtained immediately following closure of the skin in five horses (16.1%). Three of these isolates were Gram‐positive (haemolytic *Staphylococcus* spp., non‐haemolytic *Staphylococcus* spp. and *Bacillus* spp.) and two were Gram‐negative (*E. coli* and *Empedobacter brevis*) bacterial isolates. A positive intraoperative culture (following linea alba and/or skin closure) was found in nine horses (29%) (two horses had a positive culture of both linea alba and skin). Of these, four horses had received pre‐operative penicillin only while five horses had received both penicillin and gentamicin.

Fifteen horses were randomly assigned to Group A (an adhesive dressing) and 16 horses to Group B (a stent dressing). The incision was fully protected in 14 horses (45.2%) once standing in the anaesthetic recovery box, partially protected in 10 (32.3%) and was fully exposed in seven (22.6%). The proportion of horses in which the incision was classified as fully protected (7 vs. 7 horses), partially protected (3 vs. 7 horses) or fully exposed groups (5 vs. 2 horses) was similar in Groups A and B. A positive culture was obtained immediately following recovery in 11 horses (35.5%) and of these horses, there were a roughly equal proportion of horses with the incision fully protected (4 /11 horses, 36.4%), partially protected (3 /11 horses, 27.3%) and fully exposed (4/11 horses, 36.4%) irrespective of the method of incisional protection. The same bacterial types were not isolated intra‐operatively and immediately following recovery. The incision was more likely to be fully protected at the end of recovery in horses that had an excellent or good recovery with minimal ataxia (Grade 1–2/5) 17 (OR 0.08, 95% CI 0.006–0.53, P = 0.003) but there was no association between the quality of the recovery and a positive culture result (OR 0.58, 95% CI 0.09–3.22, P = 0.7).

**Figure 4:**
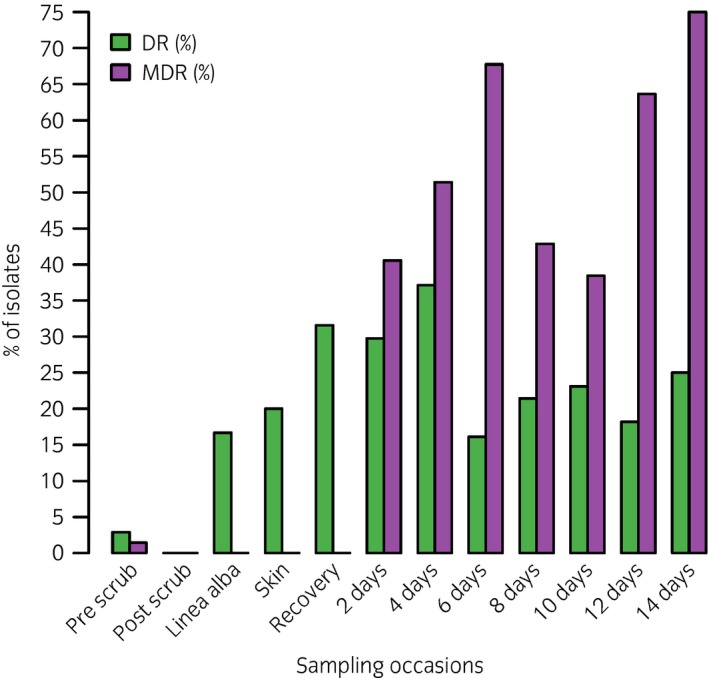
Percentage of drug resistant (DR) (resistance to ≥1 class of antimicrobials) and multi drug resistant (MDR) (resistance to ≥3 classes of antimicrobials) isolates during the study period.

At the first dressing change 2 days post‐operatively, bacteria were cultured from the incision in 19/30 horses, (60.3%) with the majority yielding a mixed growth of different bacterial species (12/19, 63.2%) whilst in seven cases (7/19, 36.8%) a pure bacterial growth was obtained. One horse was euthanised in the first 48 h following a positive ileal biopsy for equine dysautonomia and was subsequently excluded from the study. At this time point, 37 bacterial isolates were cultured from 19 horses with 37.8% (14/37) being Gram‐positive and 62.2% (23/37) Gram‐negative bacteria. The proportion of horses with a positive bacterial culture increased for the remainder of hospitalisation (75.9% at 4 days, 89.5% at 6 days, 70% at 8 days and 100% at 10 days) and the median hospital duration in this cohort of horses was 7 days (interquartile range 5–9 days). In the post‐operative period, the commonly isolated bacterial species belonged to genus *Staphylococcus* (16.2–50%). The second, third and fourth most commonly isolated bacterial species at subsequent samples were *E. coli*,* Enterococcus* spp or *Acinetobacter* spp and the proportion of each varied slightly at each time point. The proportion of both DR and MDR isolates cultured increased with increased duration of hospitalisation. The distribution of DR and MDR isolates during the study period is shown in Fig 4. Four extended spectrum beta‐lactamase (ESBL)‐producers were identified in this study: two were *E. coli*, which were obtained at 2, and 6 days post‐operatively in two different horses and the remaining two were *Kluyveria* sp. (obtained at 8 and 10 days post‐operatively in the same horse). A single methicillin‐resistant *Staphylococcus aureus* (MRSA) isolate was identified 6 days post‐operatively in one horse. No ESBL‐producers or MRSA isolates were recovered from any SSIs in the horses included in the study.

There was an overall incidence of SSI of 22.6% (7/31) during the study period; a total of three horses (9.7%) developed SSI during hospitalisation whilst SSI did not become apparent until after discharge from the hospital in four horses (12.9%), when the SSI was diagnosed by the referring veterinary surgeon. Culture results at the peri‐operative time points in the seven horses that developed a SSI are shown in Table 2. There was no significant association between SSI and any of the variables examined and the results are presented in Supplementary Items 2 and 3. The combination of penicillin and gentamicin was associated with a reduced SSI rate compared with the use of penicillin alone; however, this finding was close to but was not statistically significant (OR 0.16, 95% CI 0.02–1.36, P = 0.05).

**Table 2:** Culture results at peri‐operative time points in the seven horses that developed surgical site infection (SSI)

Horse	Linea alba	Skin closure	Following recovery	SSI culture
1	ng	ng	*Acinetobacter* spp. and *Enterococcus* spp.	*E. coli*
2	ng	ng	ng	No culture obtained
3	*Mannheimia* spp.	ng	ng	*E. coli*
4	ng	ng	ng	No culture obtained
5	*Staphylococcus* spp.	*Staphylococcus* spp.	ng	No culture obtained
6	ng	*Staphylococcus* spp.	*Staphylococcus* spp.	*Bacillus* spp.
7	*Corynebacterium* spp.	ng	*Staphylococcus* spp.	No culture obtained

No culture was obtained in those horses where SSI developed following hospital discharge. ng, no growth.

## Discussion

This is the first study to detail sequential swabbing of ventral midline incision in horses undergoing surgical management of colic. SSI following exploratory laparotomy is an important cause of morbidity and has welfare and economic consequences. The current study provides important information about the proportion of horses and the time points for which a positive bacterial culture was obtained in a hospital population. This information can be used to develop preventive strategies to minimise the risk of SSI developing.

The current study identified that effective aseptic preparation of the ventral midline had been achieved prior to surgery commencing. This is consistent with another study demonstrating that aseptic preparation, using 2% chlorhexidine gluconate scrub for at least 10 min followed by 70% isopropyl alcohol solution provides significantly more persistent antimicrobial activity at 24 h after application than either of the components used separately 22. The majority of horses in the current study had an enterotomy or anastomosis performed, increasing the likelihood of contamination of the surgical site with enteric bacteria. Despite this, the proportion of horses in which bacteria were cultured from the incision during and immediately following surgery was low, likely due to the standard hospital procedures for minimising contamination of the surgical site using appropriate surgical draping, lavage and regular changes of surgical gloves.

Prior to aseptic preparation there was a heavy mixed bacterial population on the ventral midline with a predominance of Gram‐positive (65.7%) isolates consistent with bacterial skin flora 12, 23. Post‐operatively, these bacterial populations started to be cultured from 48 h onwards, but with an overall equal split between Gram‐positive and Gram‐negative isolates. Previous studies have found a predominance of Gram‐positive isolates colonising the incision post‐operatively 11, 13, 24 (but not necessarily causing a SSI) providing support for an endogenous source of SSI, as *Staphylococcus* spp are commonly part of the resident skin flora 25. In contrast, an earlier study from our hospital found *E. coli* (59.5%) to be the most common isolate from SSIs (followed by *Enterococcus* spp. [42.4%] and *Staphylococcus* spp. [25.4%]) 26. This latter study sampled discharging SSIs, which may be more representative of isolates associated with incisional infection rather than those that colonise the skin. Previous studies have shown that SSIs in horses are usually polymicrobial in origin (40–63%) 26, 27, 28. Monomicrobial SSIs are rare and when they occur, Gram‐positive (32%) and Gram‐negative (28%) bacteria seems to be equally involved 27. The distribution of monomicrobial and polymicrobial infections are highly variable among different institutions and this has been shown in both human 29, 30 and equine studies 31. As polymicrobial cultures are commonly cultured from SSIs after gastrointestinal surgery, obtaining a representative sample which would yield a more significant bacterial culture is extremely important for a successful and targeted therapy 4. This study identified an overall low prevalence of ESBL‐producers and MRSA, but a greater prevalence of MDR isolates were cultured as the duration of hospitalisation progressed, as has been demonstrated previously 32. Identifying bacterial isolates associated with SSIs and their presence in the hospital environment form a vital part of clinical audit and is important in order to prevent future infections 4.

In the current study, a positive culture intra‐operatively or immediately following recovery did not predict development of SSI. This is similar to findings from recent studies both in dogs 33, 34, 35 and humans 36 where intraoperative contamination occurred in 12–81% canine and 46% of human orthopaedic surgeries but was not a predictor of SSI. Previous studies have identified intra‐operative contamination in 44–96% horses 12, 13 undergoing exploratory laparotomy and the culture results similarly failed to predict development of SSI. In horses where SSI developed during hospitalisation in the current study, this was due to a different bacterial isolate than that identified intra‐operatively. If this was due to contamination of the site, this may have occurred at a later stage e.g. during a dressing change but was not detected by the sampling strategy used. In addition, all horses received pre‐operative antimicrobials, which may explain why horses with a positive intraoperative culture with potentially opportunistic pathogens such as *E. coli* or haemolytic *Staphylococcus* spp, did not subsequently develop a SSI. There was an association between the use of penicillin and gentamicin and a reduced rate of SSI compared with the use of penicillin alone. This was not statistically significant but the present study was limited by the relatively small number of horses and a larger study is merited to explore this association further. A recent publication of SSIs from our hospital revealed that penicillin resistant isolates accounted for 92% of bacterial isolates while only 18% of isolates were gentamicin resistant 26. Nevertheless, antimicrobial stewardship is important and peri‐operative antimicrobial selection should be tailored to each individual case and type of surgical procedure performed 37. It is also important to highlight that the development of a SSI is not simply determined by bacterial contamination but is part of a complex interaction between the host, the pathogen and the environment and that many factors determine the outcome 38, 39.

Physical protection of the incision during recovery did not prevent SSI in the current study and in horses where the incision was not protected during recovery from anaesthesia, there was no association with either SSI or a positive culture obtained at a subsequent time point. This would also suggest that SSI is not simply related to external bacterial contamination of the incision. Another potential source of contamination of the incision was at the time of dressing change, where, despite personnel wearing clean gloves and not touching the incision, some contamination is possible. The incision was not cleaned at the time of dressing changes unless there was marked discharge from the incision, as there were concerns of introducing infection by disrupting the incisional seal. There could also have been soiling of the incision from the horses’ adjacent environment through the bandage or bandage slippage could have occurred and the incision could have been contaminated before the slippage was recognised and the dressing changed. The high proportion of positive cultures by day 4 (75.9%) is likely due to normal return of skin flora and not due to contamination. We consider the benefits of placing an abdominal bandage cannot be discounted based on these findings in a small population of horses 9.

Bacteraemia was demonstrated in a recent study during exodontia of apically infected teeth in horses and the same bacteria were cultured from the infected teeth and blood from the same horse 40. Bacteraemia has also been demonstrated in horses with colitis 41 and those undergoing surgery for management of strangulating lesions of the small intestine 42. Given the results from the current study, it is plausible that bacteraemia may explain why SSI does not simply appear to be associated with incisional contamination, and why bacteria isolated from the incision at the time of surgery did not correlate with SSI development or the bacteria subsequently isolated from incisional discharge. This is an area that requires further investigation.

Limitations of the current study include the fact that bacterial culture was limited to those that developed SSIs during hospitalisation and culture of any SSI that developed following hospital discharge would have been optimal. Despite this, intraoperative contamination was not a predictor of development of SSI in any of the cases presented. Post‐operative samples were also limited to those from the skin surface only (unless there was drainage) and are not representative of deeper tissue layers where bacteria may have been present; however, this would have likely resulted in presence of clinical signs. The virulence of the bacterial cultures was not investigated and those organisms identified in this study may pose virulence factors, which would enable them to be pathogenic or opportunistic pathogenic organisms. Future studies should attempt to classify the bacterial isolates fully to determine their pathogenic nature. The bacterial load of each sample was quantified subjectively (light, moderate, heavy) but future studies should also measure colony forming units (CFU) from tissue in an attempt to objectively quantify bacterial counts as a measure of the level of contamination. Objective enumeration may also be useful to determine the significance of isolates. The small sample size could have limited our results, which may be different in a larger study. The incidence of SSI that developed during hospitalisation in the current study was lower than the previous rate of SSI in our hospital (25.4%) 26. It is plausible the prospective nature of our study introduced an observer effect bias, the ‘Hawthorne Effect’, where members of staff may have adhered more strictly to aseptic technique as they knew they were being observed 43.

In conclusion, this study has identified that various bacterial species can be isolated both intra‐operatively and immediately following recovery without development of SSI in majority of horses. Contamination was not a predictor of development of SSI in any of the cases presented. The type of surgical procedure performed also did not influence intra‐operative culture results. The greatest proportion of bacterial cultures was at 10 days post‐operatively onwards, which likely represents bacterial recolonisation of skin. Development of SSI appears to be a multifactorial process and is not simply limited to environmental contamination of the incision. Further investigations should investigate the effect of other sources of incisional bacterial contamination such as haematogenous spread.

## Authors’ declaration of interest

No competing interests have been declared.

## Ethical animal research

Ethical approval for the study was granted by the University of Liverpool Veterinary Research Ethics Committee (VREC172). Owners gave consent for their animals’ inclusion in the study.

## Sources of funding

The cost of microbiological cultures was provided by University of Liverpool internal Veterinary Research Project Support Fund. Horse owners gave consent for their animals’ inclusion in the study.

## Authorship

C.M. Isgren, N.B. Townsend and D.C. Archer contributed to study design. C.M. Isgren contributed to study execution. D. Timofte contributed to interpretation of microbiological cultures. S.E. Salem, C.M. Isgren and D.C. Archer contributed to data analysis and interpretation. C.M. Isgren, S.E. Salem, D. Timofte, N.B. Townsend, T.W. Maddox and D.C. Archer contributed to the preparation of the manuscript. All authors gave their final approval of the manuscript.

## Supporting information


**Supplementary Item 1:** Questionnaire used during telephone interview performed at least 3 months following hospital discharge.

**Supplementary Item 2:** Descriptive statistics and a Wilcoxon rank sum test for continuous variables investigated for association with surgical site infection (SSI) in horses undergoing exploratory laparotomy. bpm, beats per minute; PCV, packed cell volume.

**Supplementary Item 3:** Descriptive statistics and a Fisher's exact test of categorical variables investigated for association with surgical site infection (SSI) in horses undergoing exploratory laparotomy. DR, drug resistant (resistance to ≥1 class of antimicrobials); MDR, multi drug resistant (resistance to ≥3 classes of antimicrobials); TB, thoroughbred; TBx, thoroughbred cross; WB, warmblood; WBx, warmblood cross.

## Manufacturers' addresses

^a^Kruuse UK Ltd, Sherburn in Elmet, North Yorkshire, UK.

^b^Deltalab, Barcelona, Spain.

^c^Biomerieux, France.

^d^TREK Diagnostic Systems, West Sussex, UK.

^e^Oxoid, Basingstoke, Hampshire, UK.

^f^Smith & Nephew Medical Ltd, Hull, Yorkshire, UK.

^g^“OrthoHorse”, VetExtras, http://www.vetextras.com.

^h^BSN Medical, Hull, Yorkshire, UK.
