# Supplementary material for: Sequential bacterial sampling of the midline incision in horses undergoing exploratory laparotomy
Source: Equine Vet J. 2018 May 17;51(1):38–44. doi: 10.1111/evj.12958 (PMC6585715; doi:10.1111/evj.12958)
Supplement: Supplementary file 1 — Supplementary Item 1: Questionnaire used during telephone interview performed at least 3 months following hospital discharge. [file EVJ-51-38-s001.pdf]

Equine Veterinary Journal

Supplementary Item 1: Questionnaire used during telephone interview performed at least 3 months following hospital discharge

Phone Number (1):

Phone Number (2):

Sequential swabbing Colic follow-up Questionnaire

Name of owner: Name of Horse:

Date of surgery: Date of follow-up:

Surgical Lesion:

Introductory statement: I am telephoning with regards to XX, your horse that underwent colic surgery at Leahurst. We are following horses after surgery to monitor their progress at home. Your participation is voluntary and may withdraw consent at any time. All information is confidential.

Would you be prepared to answer a short questionnaire? **Yes / No**
*(if **No** interview is abandoned at this stage)*

Questions regarding survival

1. Do you still own the horse? **Yes / No**
2. Is the horse still alive? **Yes / No**
3. If No: was euthanasia related to the colic/wound? **Yes / No**
4. Was euthanasia unrelated to colic/wound? **Yes / No**
5. Please specify the reason for euthanasia ________________________________
6. Date of euthanasia ___________________________

Questions regarding post-operative care following discharge:

Colic Episodes

1. Did the horse experience any further colic episodes? **Yes / No**
   -Date(s)?
2. Number of colic episodes in total?
3. Did the vet attend? **Yes / No**

Incisional Problem

4. Did the horse develop any problems with the incision? **Yes / No**
   - Drainage **Yes / No**
     - Serous / purulent
   - Oedema **Yes / No**
   - Hernia **Yes / No**
   - Did the vet attend? **Yes / No**
   - Was a swab obtained of the discharge for culture and sensitivity? **Yes / No**

Diarrhoea

5. Did the horse experience loose droppings? **Yes / No**

   - Did the vet attend? **Yes / No**

6. Did the horse experience weight loss requiring vet intervention? **Yes / No**

Questions regarding return to athletic function

7. Did the horse return to work? **Yes / No / NA**

8. What level of work was the horse in prior to colic surgery?

   - No work
   - Leisure horse (light hacking/ schooling)
   - Racing
   - Affiliated competition horse: BE/BSJA/BD/other
   - Low level/unaffiliated competition horse
   - Other (please specify)______________________

5. To what level of work has the horse returned to?

   - No work
   - Lower than previously
     - Is there a reason for lower level work?
   - Same as previously
   - Higher than previously

If competition horse, has it returned to competing? **Yes / No / NA**
