# Supplementary material for: Sequential bacterial sampling of the midline incision in horses undergoing exploratory laparotomy
Source: Equine Vet J. 2018 May 17;51(1):38–44. doi: 10.1111/evj.12958 (PMC6585715; doi:10.1111/evj.12958)
Supplement: Supplementary file 2 — Supplementary Item 2: Descriptive statistics and a Wilcoxon rank sum test for continuous variables investigated for association with surgical site infection (SSI) in horses undergoing exploratory laparotomy. bpm, beats per minute; PCV, packed cell volume. [file EVJ-51-38-s002.pdf]

Equine Veterinary Journal

Supplementary Item 2: Descriptive statistics and a Wilcoxon rank sum test for continuous variables investigated for association with surgical site infection (SSI) in horses undergoing exploratory laparotomy. bpm = beats per minute, PCV = packed cell volume.

Variable	Horses without SSI (24, 77.4%)	Horses with SSI (7, 22.6%)	P-value
Age (years)	16 (11.5-18.5)	15 (10–18)	0.8
Weight (kg)	546 (500-581.5)	546 (477.5- 581)	0.7
Heart rate on arrival (bpm)	48 (43-64)	44 (40-52)	0.4
PCV on arrival (%)	36 (33.75-43)	32 (29-35)	0.06
Total plasma protein on arrival (g/L)	68 (63.5-72)	65 (57-70)	0.4
Blood lactate (mmol/L)	1.4 (0.5-2.73)	0.9 (0.5-1.75)	0.7
Antimicrobial Duration (days)	5 (4.5- 5)	5 (4-5)	0.7
Recovery score	2 (2-3)	3 (2-3)	0.8
