# Supplementary material for: Sequential bacterial sampling of the midline incision in horses undergoing exploratory laparotomy
Source: Equine Vet J. 2018 May 17;51(1):38–44. doi: 10.1111/evj.12958 (PMC6585715; doi:10.1111/evj.12958)
Supplement: Supplementary file 3 — Supplementary Item 3: Descriptive statistics and a Fisher's exact test of categorical variables investigated for association with surgical site infection (SSI) in horses undergoing exploratory laparotomy. DR, drug resistant (resistance to ≥1 class of antimicrobials); MDR, multi drug resistant (resistance to ≥3 classes of antimicrobials); TB, thoroughbred; TBx, thoroughbred cross; WB, warmblood; WBx, warmblood cross. [file EVJ-51-38-s003.pdf]

Equine Veterinary Journal

Supplementary Item 3: Descriptive statistics and a Fisher's exact test of categorical variables investigated for association with surgical site infection (SSI) in horses undergoing exploratory laparotomy. DR = drug resistant (resistance to ≥1 class of antimicrobials), MDR = multi drug resistant (resistance to ≥3 classes of antimicrobials). TB = Thoroughbred, TBx = Thoroughbred cross, WB = Warmblood, WBx = Warmblood cross.

**Variable**	**Categories**	**Horses without SSI; 24 (77.4%)**	**Horses with SSI; 7 (22.6%)**	***Odds ratio**	**95% CI of odds ratio**	**P-value**
Sex	Female<br>Male	11 (92)<br>13 (68)	1 (8)<br>6 (32)	4.85	0.47–254	0.2
Breed	Cob/draught<br>Pony<br>TB/TBx<br>WB/WBx	7 (78)<br>6 (75)<br>5 (62)<br>6 (1)	2 (22)<br>2 (25)<br>3 (38)<br>0 (0)			0.5
Stent	No Stent<br>Stent	12 (80)<br>12 (75)	3 (20)<br>4 (25)	1.32	0.18–11.0	>0.9
Enterotomy	No Enterotomy<br>Enterotomy	6 (67)<br>18 (82)	3 (33)<br>4 (18)	0.46	0.06–4.03	0.5
Protected incision at end of recovery	Fully protected<br>Partially protected<br>Fully exposed	4 (57)<br>12 (86)<br>8 (80)	3 (43)<br>2 (14)<br>2 (20)			0.5
Antimicrobials	Penicillin only<br>Penicillin and Gentamicin	4 (50)<br>20 (87)	4 (50)<br>3 (13)	0.16	0.02–1.36	0.05
DR isolates	No DR isolate (swab 1-6)<br>DR isolate (swab 1-6)	13 (76)<br>11 (79)	4 (24)<br>3 (21)	0.89	0.11–6.58	1
MDR isolates	No MDR isolate (swab 1-6)<br>MDR isolate (swab 1-6)	19 (76)<br>5 (83)	6 (24)<br>1 (17)	0.64	0.012–7.70	1
Growth from skin prior to aseptic preparation (swab 1)	No<br>Yes	1 (50)<br>23 (79)	1 (50)<br>6 (21)	0.27	0.003–23.86	0.4
Growth from skin after aseptic preparation (swab 2)	No<br>Yes	24 (80)<br>0	6 (20)<br>1 (100)			0.2
Growth from *linea alba* (swab 3)	No<br>Yes	21 (84)<br>3 (50)	4 (16)<br>3 (50)	4.89	0.48–52.7	0.1
Growth from skin (swab 4)	No<br>Yes	21 (81)<br>3 (60)	5 (19)<br>2 (40)	2.69	0.18–31.1	0.6
Growth from skin after recovery (swab 5)	No<br>Yes	16 (80)<br>8 (73)	4 (20)<br>3 (27)	1.48	0.17–11.3	0.7

Variable	Categories	Horses without SSI; 24 (77.4%)	Horses with SSI; 7 (22.6%)	*Odds ratio	95% CI of odds ratio	P-value
Growth 48 hours post-operatively (swab 6)	No<br>Yes	9 (82)<br>14 (74)	2 (18)<br>5 (26)	1.58	0.20-20.0	>0.9
Degree linea alba growth	None<br>Light<br>Moderate<br>Heavy	21 (84)<br>1 (33)<br>1 (50)<br>1 (100)	4 (16)<br>2 (67)<br>1 (50)<br>0			0.1
Degree skin growth	None<br>Light<br>Moderate<br>Heavy	21 (81)<br>1 (50)<br>0<br>2 (100)	5 (19)<br>1 (50)<br>1 (100)<br>0			0.2
Degree skin growth following recovery	None<br>Light<br>Moderate<br>Heavy	17 (81)<br>2 (67)<br>2(100)<br>3 (60)	4 (19)<br>1 (33)<br>0<br>2(40)			0.6
Degree skin growth first dressing change (48 hrs.)	None<br>Light<br>Moderate<br>Heavy	10 (83)<br>3 (100)<br>4 (80)<br>7 (64)	2 (17)<br>0<br>1 (20)<br>4 (36)			0.7

*Odds ratio were not calculated for contingency tables containing more than 4 cells
